# Human Gastroenteritis Outbreak Associated with *Escherichia albertii*, Japan

**DOI:** 10.3201/eid1901.120646

**Published:** 2013-01

**Authors:** Tadasuke Ooka, Eisuke Tokuoka, Masato Furukawa, Tetsuya Nagamura, Yoshitoshi Ogura, Kokichi Arisawa, Seiya Harada, Tetsuya Hayashi

**Affiliations:** Author affiliations: University of Miyazaki, Miyazaki, Japan (T. Ooka, Y. Ogura, T. Hayashi);; Kumamoto Prefectural Institute of Public Health and Environmental Science, Kumamoto, Japan (E. Tokuoka, M. Furukawa, T. Nagamura, S. Harada);; University of Tokushima, Tokushima, Japan (K. Arisawa)

**Keywords:** Escherichia albertii, enteric infections, bacteria, outbreak, human gastroenteritis, Japan

## Abstract

Although *Escherichia albertii* is an emerging intestinal pathogen, it has been associated only with sporadic human infections. In this study, we determined that a human gastroenteritis outbreak at a restaurant in Japan had *E. albertii* as the major causative agent.

*Escherichia albertii* is an emerging human and bird pathogen that belongs to the attaching and effacing group of pathogens. This group of pathogens forms lesions on intestinal epithelial cell surfaces by the combined action of intimin, an *eae* gene–encoded outer membrane protein, and type III secretion system effectors (*1**–**4*).

Recently, we found that *E. albertii* represents a substantial proportion of the strains that had previously been identified as *eae*-positive *Escherichia coli*, enteropathogenic *E. coli* or enterohemorrhagic *E. coli*; 26 of the 179 *eae*-positive strains analyzed were found to be *E. albertii* (*5*). Furthermore, *E. albertii* is also a potential Shiga toxin 2f (Stx2f)–producing bacterial species (*5*). However, no *E. albertii*–associated gastroenteritis outbreak has been reported, which generates doubts regarding the clinical role of this microorganism. In this study, we revisited an outbreak of gastroenteritis that was presumed to have been caused by *eae*-positive atypical *E. coli* OUT:HNM (*6*) to determine if it was actually caused by *E. albertii*. 

## The Study

An outbreak of gastroenteritis occurred at the end of May 2011 in Kumamoto, Japan, among persons who attended 1 of 2 parties held in a Japanese restaurant on May 29. We reviewed case records for the 94 persons who attended the parties. A total of 48 persons became ill; 43 of them attended the first party (a total of 86 attended), and 5 attended the second party (a total of 8 attended). The ill participants had not eaten any food in common except for the meals served at the restaurant. The main symptoms of the patients were diarrhea (83%), abdominal pain (69%), fever (44%; mean temperature 37.2°C), and nausea (29%). The mean incubation period was 19 h.

A routine protocol to identify bacteria and viruses (Technical Appendix) was used by our laboratory to examine 54 fecal specimens from 44 party participants and 10 members of the restaurant kitchen staff (7 party participants and all of the kitchen staff were asymptomatic). Atypical *E. coli* (lactose negative; OUT:HNM) strains harboring the *eae* gene and *E. coli* OUT:H18 strains harboring the *stx2d* and *astA* (but not *eae*) genes were isolated from 24 and 3 specimen, respectively; 7 specimens yielded both strains (Table 1). The *stx2*-positive/*eae*-negative *E. coli* strains were found to be serotype O183 (a recently described O serotype) by agglutination testing with O183-specific antiserum (S. Iyoda, M. Ohnishi, unpub. data).

**Table 1:** Isolates from fecal specimens of party participants during outbreak of gastroenteritis associated with *Escherichia* *albertii*, Japan*

Isolate	Origin of isolates					
	Participants, n = 44				Kitchen staff, n = 10	
	Symptomatic	Asymptomatic	No information		Symptomatic	Asymptomatic
*E. albertii*†	21	1	0		0	2
*E. albertii*† and *E. coli* O183:H18‡	7	0	0		0	0
*E. coli* O183:H18‡	3	0	0		0	0
None	6	5	1		0	8

*None, negative for both pathogens. †Initially identified as atypical (lactose negative) *E. coli* OUT:HNM harboring the intimin (*eae*) gene. ‡Initially identified as *eae* negative *E. coli* OUT:H18 harboring the Shiga toxin 2d and enteroaggregative *E.coli* heat-stable toxin genes.

All atypical *E. coli* strains showed identical or nearly identical *Xba*I-digested DNA banding patterns by pulsed-field gel electrophoresis, and the 10 *E. coli* O183:H18 strains also exhibited identical patterns (Figure). The source of the infection was most likely the meals served in the restaurant, but a bacteriological examination of the meal or of the ingredients used to prepare the meal was not possible because none of the food was preserved for analysis.

**Figure:**
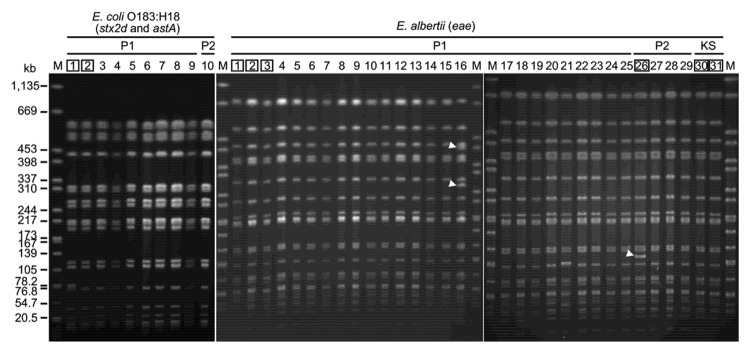
*Xba*I-digested pulsed-field gel electrophoresis profiles of isolates from fecal specimens collected from patients during an outbreak of human gastroenteritis associated with *Escherichia albertii*, Japan. Extra bands observed in 2 *E. albertii* isolates are indicated by arrowheads (only 1 or 2 band differences). The 2 *E. coli* O183:H18 and 6 *E. albertii* isolates indicated by numbers in boxes were subjected to multilocus sequence analysis (Technical Appendix). *stx2d*, Shiga toxin 2d gene; *astA,* enteroaggregative *E. coli* heat-stable toxin gene; *eae*, intimin gene. Lane M, *Salmonella enterica* serovar Braenderup strain H9812 (used as a DNA size standard); lanes P1, Party 1; lanes P2, Party 2; lanes KS, kitchen staff.

The lactose-negative/*eae*-positive features of the OUT:HNM strains suggested that these strains might be *E. albertii*. We examined additional biochemical properties of these strains and found that they exhibited the *E. albertii*–specific features described (*4**,**5*). These features include nonmotility, inability to ferment xylose and lactose, and inability to produce β-d-glucuronidase. The *E. coli* O183:H18 strains demonstrated common phenotypic and biochemical properties of *E. coli* (*7*).

To determine whether the *E. albertii*–like OUT:HNM strains were *E. albertii*, we randomly selected 6 strains and determined their phylogeny by multilocus sequence analysis as described (*5*) (Technical Appendix Table). Results indicated that although the *E. coli* O183:H18 strain analyzed in parallel belongs to *E. coli sensu stricto*, the *E. albertii*–like OUT:HNM strains belong to the *E. albertii* lineage; all 6 strains showed identical sequences (Technical Appendix Figure).

We further examined the intimin subtype by sequencing the *eae* gene, the chromosome integration site of the locus of enterocyte effacement encoding the *eae* gene and a set of type III secretion system genes, and the presence and subtype of the *cdtB* gene as described (*5*). Results showed that the *E. albertii* strains had intimin σ, which is rarely identified in enteropathogenic *E. coli* or enterohemorrhagic *E. coli*; the locus of enterocyte effacement was integrated into the *pheU* tRNA gene; and the *cdtB* gene of the II/III/V subtype group was present. These features are consistent with recently described genetic features of *E. albertii* (*5*).

We divided the party participants into 4 groups according to strain isolation patterns and statistically assessed the association of strain isolation patterns with incidence of clinical symptoms (Table 2). The results indicated that persons infected with only *E. albertii* or persons infected with *E. albertii* and *E. coli* O183:H18 had diarrhea and abdominal pain more frequently than did uninfected persons (p<0.05) and that the incidence of asymptomatic carriers was lower among persons infected only with *E. albertii*.

**Table 2:** Clinical symptoms of party participants during outbreak of gastroenteritis associated with Escherichia albertii, by pathogen identified, Japan*

Symptom	*E. albertii*, n = 21†	*E. albertii* and *E. coli* O183:H18, n = 7	*E. coli* O183:H18, n = 3	None,‡ n =11§
Diarrhea	17 (81)¶	7 (100)§	1 (33)	4 (36)
Abdominal pain	16 (76)¶	6 (86)§	2 (67)	3 (27)
Nausea	5 (24)	5 (71)§	0	1 (9)
Fever	8 (38)	4 (57)	2 (67)	4 (36)
None	1 (5)¶	0	0	5 (45)

*Values are no. (%). None, negative for both pathogens.  †One symptomatic person was excluded because no clinical record was available. ‡Negative for both pathogens. §One person was excluded because no clinical record was available. ¶A 2-tailed Fisher exact test (p<0.05) showed significant differences between the groups from which *E. albertii* or *E. coli* O183:H18 was isolated and the groups from which they were not isolated.

Nucleotide sequences obtained in this study have been deposited in the DNA Data Bank of Japan/European Molecular Biology Laboratory/GenBank database. Accession numbers and other information on sequence analyses are shown in the Technical Appendix.

## Conclusions

In this gastroenteritis outbreak, *E. albertii* or *stx2*-positive *E. coli* O183:H18 was isolated from 24 ill patients; both strains were isolated from 7 patients. Thus, although the responsible meal or food was not identified, it was most likely contaminated with these 2 microorganisms. The contribution or involvement of *E. coli* O183:H18 in this outbreak is unknown because there were 3 patients from whom only *E. coli* O183:H18 was isolated and because there were no differences in clinical symptoms between persons infected with *E. coli* O183:H18 and persons not infected (Tables 1, 2). In contrast, *E. albertii* was isolated from a larger number of patients, and many fecal specimens yielded only *E. albertii* (Table 1).

The proportion of persons who had clinical symptoms was also higher for *E. albertii*–positive party participants than for uninfected persons (Table 2). Therefore, it is plausible that *E. albertii* was the major causative pathogen of this outbreak. This information indicates that *E. albertii* can cause gastroenteritis outbreaks among humans (*5*).

More attention should be given to sporadic cases and outbreak cases caused by this emerging pathogen. It may also be informative to revisit past outbreak cases caused by *eae*-positive atypical *E. coli* if pathogens were recorded as being nonmotile, unable to ferment lactose and xylose, and unable to produce β-d-glucuronidase.

Technical Appendix Protocol used to identify bacteria and viruses in fecal specimens obtained during a human gastroenteritis outbreak associated with *Escherichia albertii*, Japan.
