# Supplementary material for: Human Gastroenteritis Outbreak Associated with Escherichia albertii, Japan
Source: Emerg Infect Dis. 2013 Jan;19(1):144–6. doi: 10.3201/eid1901.120646 (PMC3557987; doi:10.3201/eid1901.120646)
Supplement: Technical Appendix — Protocol used to identify bacteria and viruses in fecal specimens obtained during a human gastroenteritis outbreak associated with Escherichia albertii, Japan. [file 12-0646-Techapp-s1.pdf]

Article DOI: http://dx.doi.org/10.3201/eid1901.120646

Human Gastroenteritis Outbreak Associated with *Escherichia albertii*, Japan

Technical Appendix

Protocol used to identify bacteria and viruses in fecal specimens obtained during a human gastroenteritis outbreak associated with *Escherichia albertii*, Japan

Detection and Isolation of Causative Agents

We determined the causative agents for the outbreak by our routine laboratory protocol. To isolate bacterial pathogens, fecal specimens from 44 party participants and 10 members of the restaurant kitchen staff were directly placed and cultivated on the following media: deoxycholate-hydrogen sulfide-lactose (DHL) agar (Nissui Pharamceutical Co. Ltd., Tokyo, Japan) and MacConkey agar (Difco, Detroit, MI, USA) for enteric bacteria; CHROMagar Vibrio (CHROMagar, Paris, France) for the genus *Vibrio*; mannitol salt agar with egg yolk (Nissui Pharamceutical Co. Ltd.) for *Staphylococcus aureus*; NaCl glycine Kim and Goepfert agar (Nissui Pharamceutical Co. Ltd.) with egg yolk for *Bacillus cereus*; modified charcoal-cefoperazone-deoxycholate agar (Oxoid, Basingstoke, UK) for the genus *Campylobacter*; and *Clostridium welchii* egg yolk agar (Nissui Pharamceutical Co. Ltd.) with kanamycin for *Clostridium perfringens*. Bacterial colonies were grown on DHL and MacConkey agar plates (33 specimens).

Five colonies (including white and red colonies when both were present) were picked from each of the DHL agar plates and subjected to PCR for detection of pathogenic *Escherichia coli* marker genes. Species were identified by using the Api20E System (bioMérieux, Lyon, France).

For virus investigations, 5 fecal specimens were randomly selected from 37 patients (symptomatic persons) and subjected to reverse transcription PCR to detect norovirus, sapovirus, rotavirus, adenovirus, astrovirus, and kobuvirus, according to described protocols (*1*). Results were negative for all 6 viruses whose presence was assessed.

PCR Detection of Pathogenic *E. coli* Marker Genes

PCR screening was performed for 9 pathogenic *E. coli* marker genes: *stx1*, *stx2*, *invE, eae*, *bfp*, *aggR*, *astA*, the heat-labile enterotoxin gene, and the heat-stable enterotoxin gene. All primers used for screening have been described (*2–4*). KAPATaq EXtra DNA polymerase (KAPA Biosystems, Inc., Woburn, MA, USA) was used for PCR amplification.

DNA Sequencing and Subtype Determination of the *stx2* Gene

The *stx2* gene of *E. coli* O183:H18 was amplified by using primers 5′-GATGGCGGTCCATTATC-3′ (*5*) and 5′-CGCCATAAACATCTTCTTCA-3′, which were designed on the basis of the nucleotide sequence of a highly conserved region in the gene encoding Stx2 subunit B, and KAPATaq EXtra DNA polymerase. The nucleotide sequence of the PCR product was determined by direct sequencing of the amplicon by using an ABI 3710 Autosequencer (Life Technologies, Carlsbad, CA, USA) with primers used for PCR amplification. The subtype of the *stx2* gene was determined by using a blastx homology search against known *stx2* sequences (www.ncbi.nlm.nih.gov/BLAST/).

Sequencing of Other Genes and Nucleotide Sequence Accession Numbers

In addition to the *stx2* gene of *E. coli* O183:H18, we determined the sequences of the *eae* and *cdtB* genes and 7 housekeeping genes (*adk*, *fumC*, *gyrB*, *icd*, *mdh*, *purA*, and *recA*) of 6 *E. albertii* strains and the same 7 housekeeping genes of 2 *E. coli* O183:H18 strains as described (*6*). These 8 strains were randomly selected and are indicated by boxed numbers in the Figure in the main text.

Because nucleotide sequences of these genes were identical among the 6 *E. albertii* strains and between the 2 *E. coli* O183:H18 strains, sequences of the *E. albertii* strain KU20110014 and *E. coli* O183:H18 strain KU2011009 have been deposited in the DNA Data Bank of Japan/European Molecular Biology Laboratory/GenBank database. Both strains were isolated from the same patient, who had diarrhea and abdominal pain. Accession numbers of the deposited sequences are AB714729 (*eae* of KU20110014), AB714730 (*cdtB* of KU20110014), AB741082 (*stx2d* of KU2011009), and AB714731-AB714744 (*adk*, *fumC*, *gyrB*, *icd*, *mdh*, *purA*, and *recA* of KU20110014 and KU2011009).

References

1. Harada S, Okada M, Yahiro S, Nishimura K, Matsuo S, Miyasaka J, et al. Surveillance of pathogens in outpatients with gastroenteritis and characterization of sapovirus strains between 2002 and 2007 in Kumamoto Prefecture, Japan. J Med Virol. 2009;81:1117–27. PubMed http://dx.doi.org/10.1002/jmv.21454

2. Sueyoshi M, Fukui H, Tanaka S, Nakazawa M, Ito K. A new adherent form of an attaching and effacing *Escherichia coli* (*eaeA*+, *bfp*-) to the intestinal epithelial cells of chicks. J Vet Med Sci. 1996;58:1145–7. PubMed http://dx.doi.org/10.1292/jvms.58.11_1145

3. Kobayashi K, Seto K, Yatsuyanagi J, Saito S, Terao M, Kaneko M, et al. Presence of the genes regarding adherence factors of *Escherichia coli* isolates and a consideration of the procedure for detection of a diarrheagenic strain. Kansenshogaku Zasshi. 2002;76:911–20. PubMed

4. Taguri T, Noguchi H, Hirayama H. The simultaneous detection method of 18 species of food-borne pathogenic bacteria by multiplex PCR. Annual Report of Nagasaki Prefectural Institute of Public Health and Environmental Sciences. 2002;48:43–56.

5. Paton AW, Paton JC, Manning PA. Polymerase chain reaction amplification, cloning and sequencing of variant *Escherichia coli* Shiga-like toxin type II operons. Microb Pathog. 1993;15:77–82. PubMed http://dx.doi.org/10.1006/mpat.1993.1058

6. Ooka T, Seto K, Kawano K, Kobayashi H, Etoh Y, Ichihara S, et al. Clinical significance of *Escherichia albertii*. Emerg Infect Dis. 2012;18:488–92. PubMed http://dx.doi.org/10.3201/eid1803.111401

Technical Appendix Table. Reference strains used in multilocus sequence analysis of fecal specimens obtained from party participants during outbreak of gastroenteritis associated with *Escherichia albertii*, Japan

Bacteria, strain name (serotype)	Reference or accession no.
Escherichia albertii	
Bird_2	Ooka et al. (*6*)
Bird_3	
Bird_5	
Bird_8	
Bird_13	
Bird_16	
Bird_23	
Bird_24	
Bird_25	
Bird_26	
EC03–127	
EC03–195	
EC05–44	
EC05–81	
EC05–160	
EC06–170	
24	
94389	
20H183	
20H38	
4051–6	
CB10113	
CB9786	
CB9791	
HIPH08472	
E2675	
LMG20976	ABKX00000000
E. coli	
Sakai (EHEC O157:H7)	BA000007
11368 (EHEC O26:H11)	AP010953
11128 (EHEC O111:H-)	AP010960
12009 (EHEC O103:H2)	AP010958
K-12 MG1655	U00096
HS (O9)	CP000802
SE11	AP009240
SE15 (O150:H5)	AP009378
E24377A (ETEC O139:H28)	CP000800
B171 (EPEC O111:H-)	AAJX02000100
E2348/69 (EPEC O127:H6)	FM180568
O6:K2:H1, CFT073	AE014075
UTI89 (UPEC)	CP000243
APEC (O1:K1:H7)	CP000468
Shigella sonnei	
Ss046	CP000038
S. boydii	
Sb227	CP000036
BS512 CDC 3083–94	CP001063
S. flexneri	
2a 2457T	AE014073
2a 301	AE005674
S. dysenteriae	
Sd197	CP000034
E. fergusonii	
UMN026	CU928163
Salmonella enterica serovar Typhi	
CT18	AL513382

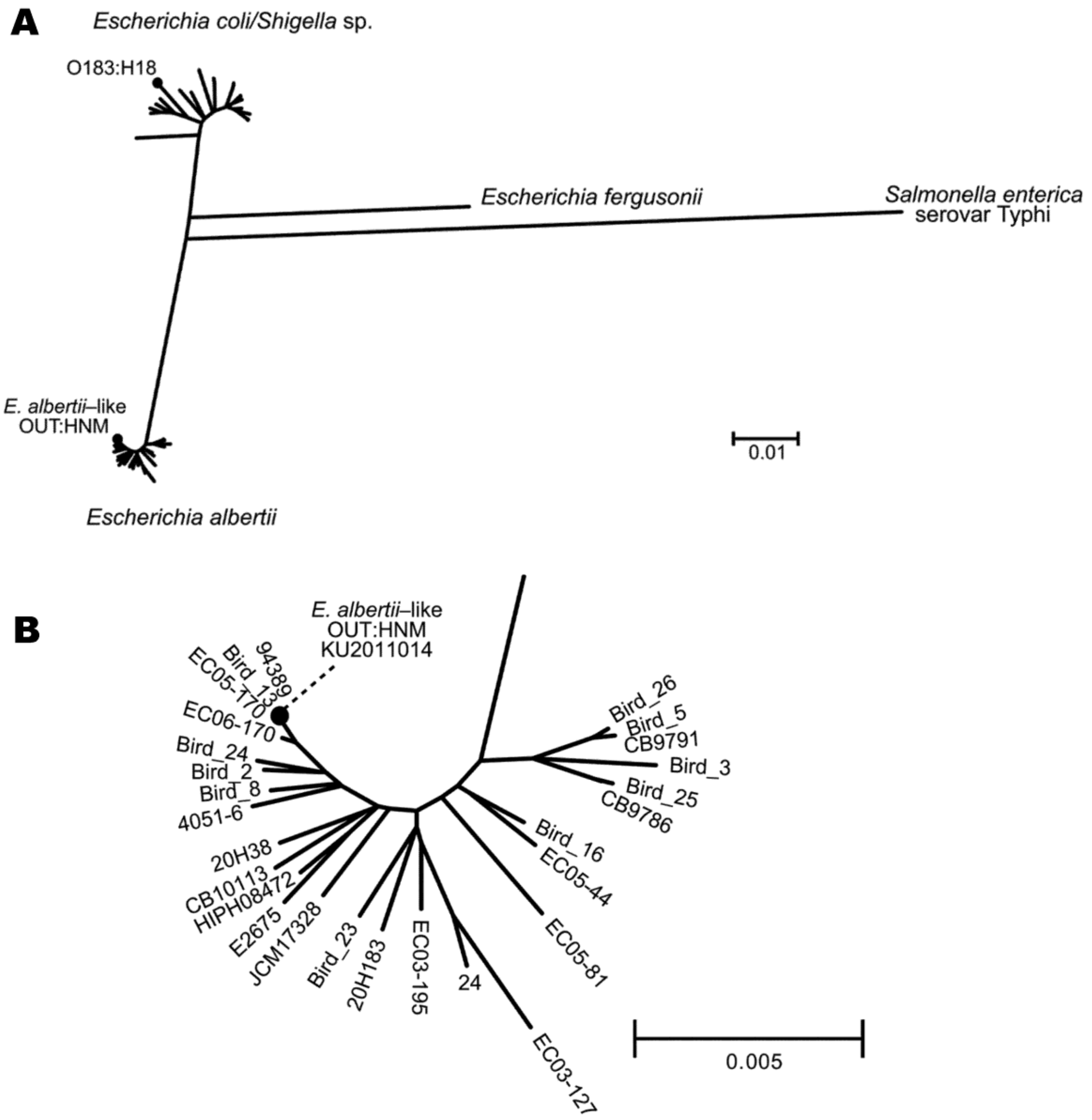

Technical Appendix Figure. A) Phylogenies of the *Escherichia albertii*–like OUT:HNM and *E. coli* O183:H18 strains determined by multilocus sequence analysis. Neighbor-joining tree constructed with concatenated partial nucleotide sequences of 7 housekeeping genes. The 49 strains (27 *E. albertii*, 20 *E. coli*/*Shigella* sp., 1 *E. fergusonii*, and 1 *Salmonella enterica* serovar Typhi) are included as references (online Technical Appendix Table). B) Enlarged view of the *E. albertii* lineage. Scale bars indicate nucleotide substitutions per site.
